# Comparison between data obtained through real-time data capture by SMS and a retrospective telephone interview

**DOI:** 10.1186/1746-1340-18-10

**Published:** 2010-05-26

**Authors:** Bendt Johansen, Niels Wedderkopp

**Affiliations:** 1 The Department of Research, the Spine Center, Hospital Lillebaelt, Oestre Hougvej 55, 5500 Middelfart, Denmark; 2 Institute of Regional Health Services Research, University of Southern Denmark, Winsloewparken 19.3, 5000 Odense, Denmark

## Abstract

**Background:**

The aims of the current study were: a) to quantitatively compare data obtained by Short Message Service (SMS) with data from a telephone interview, b) to investigate whether the respondents had found it acceptable to answer the weekly two SMS questions, c) to explore whether an additional weekly third SMS question would have been acceptable, and d) to calculate the total cost of using the SMS technology.

**Methods:**

SMS technology was used each week for 53 weeks to monitor 260 patients with low back pain (LBP) in a clinical study. Each week, these patients were asked the same two questions: "How many days in the past week have you had problems due to LBP?" and "How many days in the past week have you been off work due to LBP problems?" The last 31 patients were also contacted by telephone 53 weeks after recruitment and asked to recall the number of days with LBP problems and days off work for the a) past week, b) past month, and c) past year. The two sets of answers to the same questions for these patients were compared. Patients were also asked whether a third SMS question would have been acceptable. The test-retest reliability was compared for 1-week, 1-month, and 1-year. Bland-Altman limits of agreement were calculated. The two quantitative questions were reported as percentages. Actual costs for the SMS-Track-Questionnaire (SMS-T-Q) were compared with estimated costs for paper version surveys.

**Results:**

There was high agreement between telephone interview and SMS-T-Q responses for the 1-week and 1-month recall. In contrast, the 1-year recall showed very low agreement. A third SMS question would have been acceptable. The SMS system was considerably less costly than a paper-based survey, beyond a certain threshold number of questionnaires.

**Conclusion:**

SMS-T-Q appears to be a cheaper and better method to collect reliable LBP data than paper-based surveys.

## Background

### Different methods of data collection

The quality of clinical research depends to a large degree on the veracity of data obtained directly from patients. There are various methods that can be used to collect data, such as personal interview, observation, and questionnaires. Data collection techniques can utilize different technologies, either singly or in combination. Questionnaire data can be collected on paper, on a computer, or be Internet-based. Each method has its advantages and disadvantages. Most data are collected with a view to the past, and prospective studies are, in fact, usually a consecutive number of snap-shots in which retrospective data are collected, in order to try to describe a continued process across time. A problem with this method is that people do not always pay attention to, or remember, what researchers want them to report, and therefore these snap-shots may be inaccurate, especially when trying to remember events that occurred some time ago.

#### Traditional data collection

##### Questionnaires

Questionnaires are useful when studying a large number of people and they have several advantages. In relation to the respondents, no prior arrangements are needed, and questionnaires are familiar to many people. In the case of embarrassing questions, questionnaires are better than face-to-face or telephone interviews, and the respondent has time to reflect on the response and can also choose to remain anonymous [1]. The main disadvantage is that the literacy level of adults, which governs their ability to understand, use and reflect on written text, is inadequate amongst 10% to 50% of the populations in Europe [2]. Also, several mail-outs of the questionnaires are often required in order to achieve a reasonable response rate, respondents may misunderstand or fail to respond to individual questions, and the data must be entered into an electronic data file before analysis can take place. This makes paper questionnaire surveys a labour-intensive method and therefore, rather expensive and time consuming.

On the other hand, web-based questionnaires are inexpensive and responses can be recorded directly into a data file. However, this method of data collection requires the respondents not only to have access to a personal computer and the Internet but also to be computer-literate and computer-active. In 2003, the proportion of people in this category was estimated to be between 20% and 60% in Europe [2].

##### Face-to-face and telephone interviews

Personal interviews can be undertaken on a one-to-one or group basis. They have several advantages. Because of the personal contact, a good response rate is likely and the response is immediate [3]. The telephone interview is a cheap alternative to the personal, face-to-face interview. It is quick, has a high response rate and the interviews can continue until the required number of respondents is achieved. The disadvantages are that the participant's telephone number must be known and it is not always easy to obtain a response to telephone calls. Also obsequiousness bias might arise if respondents have a tendency to please or impress, create a false personal image, or end the interview quickly.

##### Diary

Diaries make it possible to gather longitudinal information at short time intervals about the way individuals feel or spend their time on certain activities of relevance to a research project - for example compliance with treatment, nature of lifestyle, or change in symptoms over time. The diarists need to be of a certain educational level, clear about what they are being asked to record, and comfortable with what the researcher plans to do with the data [1].

##### SMS - a novel method to collect data

Recently, an alternative method of data collection has become available in the form of 'Short Message Service' (SMS). SMS monitoring should be situated in the landscape of Ecological Momentary Assessment (EMA) described by Schiffman [4] as technology that collects real world information in real time about a patient's current state. In EMA, assessments are made frequently over time. A metaphor is used comparing EMA to recording a video-documentary giving a more detailed impression over time and across situations. A single still picture supposedly representing the true value or event over time will not be as informative as the video. Since EMA was introduced in 1994, different technologies have been used to achieve these frequent assessments, such as diaries, interviews, Personal Digital Assistants and lately also SMS. The current SMS technology combined with the necessary software avoids the potential problems with paper diaries being falsified by patients backfilling the diaries, since the patient responses are tagged with time information in the server storing the information.

The main areas in which EMA using SMS-technology has been used until now have been the monitoring of alcohol use [5], smoking cessation [3,6], physical activity, anti-obesity behaviour, and blood sugar levels [7]. SMS has many advantages and is accessible for most people. For example, in the first half of 2009, 6.5 billion SMS were sent globally, which is 6 SMS per Dane per day [8]. This technology makes it possible to deliver a short message directly to nearly every person regardless of time, place or setting.

##### Requirements for data collection with SMS

With the appropriate software, the researcher can access the SMS captured data via the Internet during the data collection phase. This makes it possible to directly identify non-responders and recognize misunderstandings. Non-responders can therefore be contacted to rectify any misunderstandings and improve compliance. The advantages are that there is no interviewer bias, that the questions are quickly answered and returned compared with frequent mail-outs of questionnaires with stamped envelopes, because the data are automatically transferred to an electronic data file that can be accessed directly for analysis. Such a system, the SMS-T-Q [9], was used in a research project that formed the basis to the current study.

#### Aims

This study had three aims. The first aim was to quantitatively compare data obtained every week for 53 weeks using SMS-T-Q with data from a telephone interview. This interview was conducted at Week 53, asking the same questions as in the SMS-T-Q survey. The recall periods for the telephone interviews were 1 week, 1 month and 1 year. Secondly, we wanted to find out whether an additional SMS question every week would have been acceptable. The third aim was to compare the total cost of using the SMS-T-Q technology with standard posted questionnaires.

## Materials and methods

### Design

In the current study we obtained data via a telephone interview and compared these with data obtained by SMS-T-Q.

#### Study subjects

The patients came from a sample of consecutive patients referred by chiropractors, medical doctors and medical specialists to The Spine Centre of Southern Denmark. To be included in the study, the patients had to have been diagnosed as having low back pain (LBP), where back pain dominated over any leg pain. Any serious pathology would exclude participation. Also, they should have been on sick leave due to their back pain some time during the past year. Unemployment was accepted but had to be due to the current LBP. Patients in the previous study participated in a randomized controlled trial with a follow-up period of 1 year. Patients in the current study however were the last 31 patients. Attempts were made to contact these 31 persons by telephone in Week 53, just after they had returned the last answer by SMS in the trial.

Written informed consent was obtained from each patient at baseline according to regulations from the Danish Data Protection Agency. A copy of the written consents is available for review by the Editor-in-Chief of this journal.

### Questions and comparisons of interest

Comparisons were made between the answers to two quantitative questions asked using the two different data collection methods, the SMS-T-Q and telephone interview. These questions were "How many days in the past week have you had problems due to LBP?" and "How many days in the past week have you been off work due to LBP problems?" 'Problems due to LBP, as it is used in our study, has a similar conceptual basis as 'bothersomeness' explained by Dunn and Croft [10]. The word 'problems' was intended to serve as a simple summary of outcomes for specific symptoms. At the time of recruitment, patients were informed about what 'problems' were supposed to cover, for example pain, stiffness and discomfort.

One version of the answers came from data obtained by the SMS-T- Q. Each week for 53 weeks these questions were sent by SMS. The patients were asked these questions only with respect to the last week, and they were instructed to use their phone's 'answer the SMS' menu button, to press a number between 0 and 7 for the number of days relevant for the answer, and to activate the 'send' button. Ten seconds after the central server registered the answer to the first question, the second question was sent to the patients. Thus, the process of answering took approximately 30 seconds per question.

The second version of the answers came from the telephone interview. Patients were asked 53 weeks after inclusion in the previous study to recall the number of days having had LBP-problems and being sick-listed for 1 week, 1 month, and 1 year. The answer from 1-week recall by telephone was compared with the same week obtained by SMS. The answer for the 1-month recall by telephone was compared with 1 month by SMS by aggregating data from the corresponding 4 weeks obtained each week by SMS. The answer for the 1-year recall by telephone was compared with 1 year by SMS by aggregating data from the corresponding 52 weeks obtained each week by SMS.

In addition, during the telephone interview, the patients were asked whether a third SMS question per week would have been acceptable, such as asking about the severity of the problems due to LBP.

With respect to the analysis of cost we added the SMS-T-Q license and the cost of the SMS. The estimate of the cost for the same volume of questionnaires as the number of SMS sent to the patients is based on the research secretaries' estimate to process one questionnaire with an additional 40% for non-responders.

### Test-retest reliability

We calculated the test-retest reliability using the two different answers for each of the three time intervals to the same questions about LBP problems and sick leave. Reliability is "a fundamental way of reflecting the amount of error both random and systematic, inherent in any measurement " [11]. In relation to the 1-week, 1-month and 1-year interval, we calculated proportions of agreement and Bland-Altman limits of agreement. Stata 10 was used for analysis.

In relation to proportions of agreement, responses that involved 1-week recall had to be identical to be considered acceptable. With respect to the 1-month and 1-year recall, less stringent criteria were applied due to the longer periods of recall. In relation to the 1-year recall there were some missing answers obtained via SMS-T-Q over the 1-year trial period. For example, if one of the weeks was missing, the sum of the data was expressed as a plausible range.

Firstly, this range was extended from the lowest value to the highest value possible given the known responses. This was because if data from one week were missing, that response, had it been obtained, could have been any number between 0 and 7. Therefore, the plausible total for the whole year could have ranged from the sum of the obtained week's data plus 0 for the missing weeks to the sum of obtained weeks plus 7 times the number of missing weeks.

Secondly, since it would be difficult for some patients to remember the exact number of days and some patients had a variable response from week to week, we also increased the tolerance for the 1-month and 1-year recall periods. Adding and subtracting two standard deviations to the results achieved this. Furthermore, we constructed an alternative data set for the one-year period where missing data were substituted by the mean value of the existing data. This was done to be able to use the Bland-Altman calculations for the average difference between measurements.

## Results

### Patient characteristics

We were able to make contact with 25 of the 31 patients (81%) for a telephone interview. As one can see in Table 1, the contactable patients were not significantly different with respect to age, LBP-score, disability-score, depression-score and psychosocial-score. These baseline data were collected in the previous study. Due to time constraints inherent in the study, no further attempts were made to contact the non-responders beyond the 1-week limit.

**Table 1:** Comparison of baseline characteristics between patients who could and could not be contacted

		Contactable patients, n = 25	Non contactable patients, n = 6	p value for differences of mean between patients contactable and not contactable
Age	Mean	41	37	.3
		
	Min	26	21	
		
	Max	58	50	

LBP according to Low Back Pain Rating Scale (Range 0-30)	Mean	19	17	.2
		
	Min	10	11	
		
	Max	26	24	

Disability according to Low Back Pain Rating Scale (Range 0-100%)	Mean	52	45	.4
		
	Min	23	19	
		
	Max	80	79	

Depression score according to Beck Depression Inventory	Mean	10	12	.5
		
	Min	1	4	
		
	Max	28	28	

Psychosocial score according to Orebro Musculoskeletal Pain Screening Questionnaire	Mean	115	104	.4
		
	Min	69	52	
		
	Max	154	161	

### Agreement between the telephone interview and SMS-Track-Questionnaire

In Table 2, we can see the proportions of patients with matching answers in the telephone interview compared with the SMS values obtained by SMS-T-Q and the Bland-Altman calculations for average differences, in number of days, between measurements.

**Table 2:** Differences between the two data-obtaining methods

Differences across time in proportions of agreement in the two different data capture methods			
	Week compared to month comparing SMS-T-Q to telephone interview	Week compared to year comparing SMS-T-Q to telephone interview	Month compared to year comparing SMS-T-Q to telephone interview

Q1: How many days have you had problems due to LBP?	22/25 compared to 19/25: Difference is 12% (95% CI: -9:33), p = .27	22/25 compared to 10/25: Difference is 48% (95% CI: 25:71), p = .000	19/25 compared to 10/25: Difference is 36% (95% CI:11:61), p = .01

Q2: How many days have you been on sick leave due to the LBP problems?	24/24 compared to 22/25: Difference is 12% (95% CI: -0,7:25), p = .08	24/24 compared to 12/25: Difference is 52% (95% CI:32:72), p = .000	22/25 compared to 12/25: Difference is 40% (95% CI: 17:63), p = 0.002

Bland-Altman average difference between measurements with 95% limits of agreement			

	1 week recall	1 month recall	1 year recall

Q1: Average difference between the two different data capture methods	0.1 days (-1 day: 0.9 day)	0.7 days (-4 days: 5 days)	36 days (-175 days: 103 days)

Q2: Average difference between the two data capture methods	0.0 days	0.3 days (-3 days: 2.5 days)	26 days (-67 days: 119 days)

In relation to the proportions of agreement, the test-retest reliability is equally good for the two questions. Across time there are significant differences in proportions of agreement when we compare week proportions to year proportions and month proportions to year proportions, with the differences in proportions ranging from 36% to 48%. However, there is no significant difference in the week to month comparisons.

With respect to the Bland-Altman limits of agreement, there is less than a day's difference in relation to the 1-week and 1-month recall periods. In relation to 1 year, the differences increased many fold to an average difference of 36 days for question 1 and 26 days for question 2.

A post hoc analysis revealed that patients with LBP problems or sick leave either every day or none of the days, could easily reproduce their previous SMS answers in the telephone interview. Those with 'in between' answers were much less able to do so (data not shown).

### Patients' acceptance of a third SMS question

The results from the telephone interview show that all of the 25 contactable participants (95% CI = 87 to 100%) thought it was acceptable to have to answer an additional third SMS question.

### Cost incurred and comparison with questionnaire survey

With respect to the price for running an SMS-system, the program is leased and hosted on a server for each project. For the previous study (n = 260), the lease was 8700 EUR including VAT. Thus there is a basic cost to consider that has to be paid up-front. The price for sending the SMS messages was 830 EUR. So, the cost in total was 9530 EUR.

The cost of following a patient by questionnaires is quite high. According to our research secretaries, the time used per questionnaire per patient would be 15 minutes + 40% extra for reminders to non-responders, thus, approximately 20 minutes per patient. The cost for this method would obviously depend on the level of salaries, but in Denmark this would amount to approximately 9 EUR per questionnaire including stamps. As a comparison, you could therefore only follow 20 patients per week for 1 year for the same cost of 9530 EUR.

## Discussion

This was a study in which two methods of data collection was compared for two LBP variables: days with problems and days with sick leave - both due to LBP.

The results of this study showed that agreement between the two methods was high for 1-week recall and 1-month recall. However the test-retest reliability declined to a significantly less acceptable level when the recall period was 1 year. Similar findings were noted by Severens, where the percentages for matching answers decreased from 95% at the 1-week comparison to 51% after one year [12].

It seems that memory loss is less pronounced in retrospective reports when events are distinct and important like no days at all with sick leave or sick leave every day. Variance in the number of days across weeks is more likely to increase the memory loss. This is in accordance with the literature on this subject [13].

Our study showed that 3 questions would have been acceptable. This is also in agreement with previous observations [4].

Providing that a minimum number of text messages are sent, the cost of gathering weekly data is considerably cheaper than the time consuming mail-out questionnaires. Because there is a basic cost of the SMS system, it cannot compete if less than a certain number of questionnaires are needed. Above this threshold, it becomes increasingly cheaper.

Although our study sample was modest, we consider our data to be unbiased. The information was derived from a sample of the last 31 of 261 participants included in a study where participants were followed each week for 53 weeks by SMS-T-Q. It was possible to make contact with 25 patients from the 31 participants (81%) within the required 5 days of answering the last SMS-questions in the previous study. Failure to obtain answers from the missing 6 people was caused by the time constraints of the study rather than particular traits with respect to the patients.

## Conclusion

Retrospective data can safely be collected for up to one month. Beyond that time span, recall becomes imprecise. The SMS-T-Q was found to be a practical, cheap and well-accepted method to collect answers to regular brief questions and would therefore be a suitable alternative to retrospective surveys. Our project showed good test-retest reliability between data from the two different measurement methods for 1 week and 1 month time intervals. For periods above 1 month, SMS-T-Q should be considered.

The cost of running the system is very low compared with postal questionnaires when more than a certain amount of data capture is needed.

## Abbreviations

EUR: Euro; LBP: Low Back Pain; Q1: Question 1; Q2: Question 2; SMS: Short Message Service; SMS-T-Q: SMS-Track-Questionnaire

## Competing interests

The authors declare that they have no competing interests.

## Authors' contributions

Both authors discussed the idea, aim and design of the investigation. BJ performed the telephone interviews, did the analyses, and wrote the manuscript. Both authors read and approved the final version of the manuscript. We are grateful to professor Charlotte Leboeuf-Yde for advice on the method and final manuscript.
